# Supplementary material for: Causal interplay between lactose intolerance and gut microbiota: a combined bidirectional Mendelian randomization and in vivo validation study
Source: Front Nutr. 2026 Jun 1;13:1803337. doi: 10.3389/fnut.2026.1803337 (PMC13265576; doi:10.3389/fnut.2026.1803337)
Supplement: Supplementary file 1 [file Data_Sheet_1.zip › supplementary materials/Forward/forest plot/ebi-a-GCST90027734.finngen_R12_E4_LACTONAS.pdf]

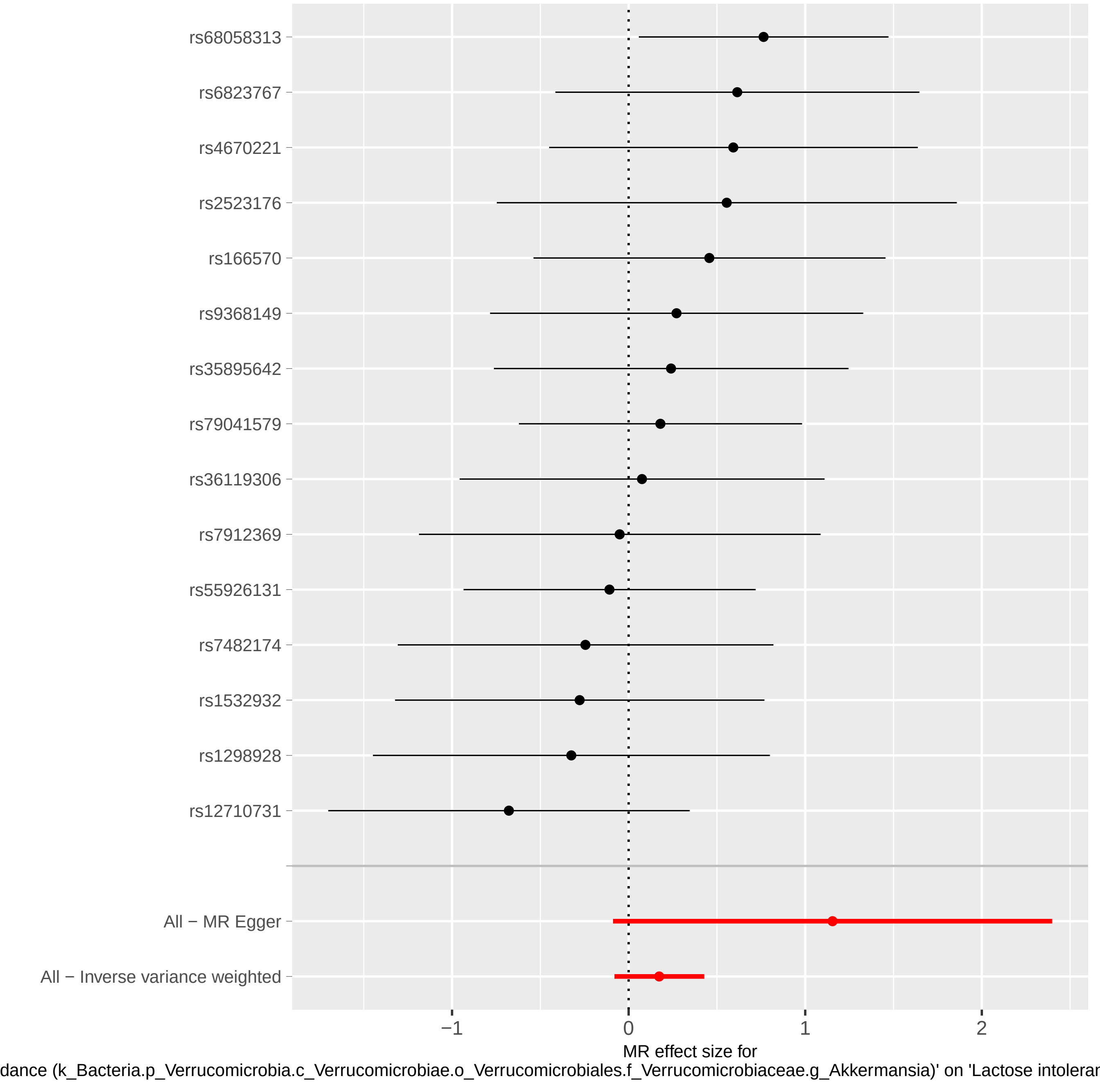
rs68058313
rs6823767
rs4670221
rs2523176
rs166570
rs9368149
rs35895642
rs79041579
rs36119306
rs7912369
rs55926131
rs7482174
rs1532932
rs1298928
rs12710731
All − MR Egger
All − Inverse variance weighted
−1
0
1
2
MR effect size for
dance (k_Bacteria.p_Verrucomicrobia.c_Verrucomicrobiae.o_Verrucomicrobiales.f_Verrucomicrobiaceae.g_Akkermansia)' on 'Lactose intoleran
